# Comparing the genetic and serum expression of LL-37 Antimicrobial Peptide in pemphigus vulgaris and pemphigus foliaceus patients^☆^

**DOI:** 10.1016/j.abd.2025.501262

**Published:** 2026-01-15

**Authors:** Fatma Dhaffouli, Nesrine Elloumi, Khadija Sellami, Emna Bahloul, Safa Tahri, Hamida Turki, Hend Hachicha, Olfa Abida

**Affiliations:** a Department of Immunology, Research Laboratory “Autoimmunity, Cancer and Immunogenetics” (LR18SP12), Habib Bourguiba Hospital, University of Sfax, Sfax, Tunisia; b Department of Dermatology, Hedi Chaker Hospital, University of Sfax, Sfax, Tunisia

Dear Editor,

Pemphigus is an immunobullous disorder characterized by flaccid blisters and erosions of skin/mucous membranes with high morbidity if left untreated. Infections: resulting from fragile skin barrier, dysfunction of immunity, and systemic corticosteroid use, are the most frequent complications of patients with pemphigus and account for 22.6% of all deaths.^1^ To cope with the microbial exposure, cells produce several Antimicrobial peptides (AMPs) which inhibit the invasion of pathogens. LL-37 is the unique cathelicidin peptide that has piqued the interest of the research community because of its numerous immune system-modulating properties.^2^ Indeed, dysregulation of LL-37 is associated with the onset and progression of multiple autoimmune diseases.^3^ The scarcity of studies in autoimmune bullous diseases was the rationale for the design of our study, aiming to offer a concise general view of the expression of LL-37 and briefly discuss the role of this small peptide as a key factor in the development of pemphigus.

This study enrolled pemphigus foliaceus (PF) and pemphigus vulgaris (PV) patients, recruited at the Dermatology Department in the Hedi Chaker University Hospital of Sfax, Tunisia, as detailed in the flow chart (Fig. 1). Pemphigus diagnosis is confirmed according to international recommendations. Patients were classified, according to disease stage and treatment management, into 3 groups: newly diagnosed, untreated and treated patients with remittent pemphigus (PDAI ≤ 8) or chronic patients’ group (PDAI ≥ 9). The LL-37 mRNA expression in PBMC was assessed in 20 PV and 27 PF patients in comparison to 16 Healthy Controls (HC), by Quantitative-PCRusing Gene-specific primers (F: 5’-TCGGATGCTAACCTCTACCG-3’/R: 5’-GGGTACAAGATTCCGCAAAA-3’) and normalized to the average housekeeping gene GAPDH. Serum concentration of LL-37 was measured with a Human Antibacterial Protein LL-37 ELISA Kit from CUSABIO (CSB-EL004476HU). Data were analyzed with SPSS software 2.0, using adapted tests. A summary of demographic, clinical, and serological data is presented in Table 1.

**Figure 1:**
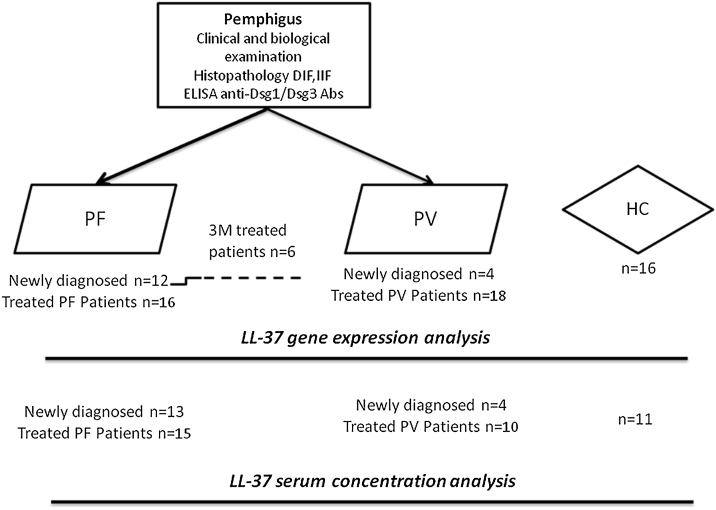
Flowchart for the studied groups. DIF, Direct Immunofluorescence; Dsg, Desmoglein; IIF, Indirect Immunofluorescence; PF, Pemphigus Foliaceus; PV, Pemphigus Vulgaris; HC, Healthy Control.

**Table 1:** Demographic, clinical and serological characteristics of pemphigus patients.

	Pemphigus Foliaceus patients (n = 29)		Pemphigus Vulgaris patients (n = 22)	
	Newly diagnosed patients (n = 13)	Treated Patients (n = 16)	Newly diagnosed patients (n = 4)	Treated Patients (n = 18)
**Age mean**	40	55	45	54
**Sex ratio M/F**	2/12	0/16	1/4	6/18
**Disease duration (year)**	‒	7.6	‒	4.7
**Treatment**	‒	45.8% Corticosteroids	‒	41.6% Corticosteroids
		54.2% Corticosteroids + Immunosuppressors		58.4% Corticosteroids + Immunosuppressors
**PDAI score mean**	34.09	17.74	44.67	15.19
**Anti-Dsg1**	137.47 U/mL	93.3 U/mL	71.5 U/mL	56.13 U/mL
**Anti-Dsg3**	1.81 U/mL	2.4 U/mL	151.5 U/mL	72.9 U/mL

Although PF and PV are clinically distinct, they share common immunological mechanisms. In our dataset, the biological markers and clinical parameters under investigation did not differ significantly between the two forms. Overall, the LL-37 expression profile was differentially dysregulated in pemphigus. LL-37 gene as well as serum expressions were significantly up-regulated in pemphigus patients compared to HC (Fig. 2A‒B). In detail, LL-37 mRNA gene expression was significantly enhanced in newly diagnosed PV and PF compared to HC (p = 0.012, p = 0.048; respectively) (Fig. 2A).

**Figure 2:**
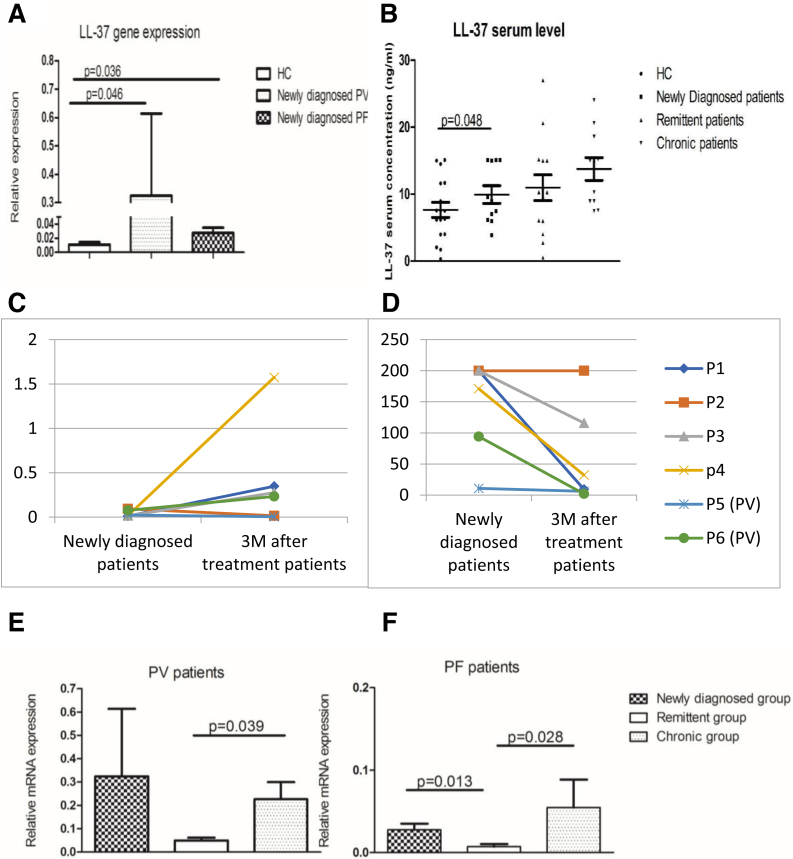
**LL-37 expression change in different studied groups.** (A) Differential expression level of LL-37 in newly diagnosed pemphigus patients (PV; n = 4, PF; n = 11) compared to HC (n = 16); (PV_mean_ = 0.324 ± 0.28, PF_mean_ = 0.027 ± 0.007 and HC_mean_ = 0.011 ± 0.003, with p = 0.046, p = 0.036; respectively). (B) LL-37 serum concentration comparison between Newly diagnosed patients (n = 15) and HC (n = 11) (Newly diagnosed patients_mean_ = 10.06 ± 1 ng/mL, HC_mean_ = 6.22 ± 1.3 ng/mL, p = 0.048) and between remittent patients (n = 11) and chronic patients (n = 14) (Remittent patients_mean_ = 9.5 ± 1.8 ng/mL, chronic patients_mean_ = 14.2 ± 1.7 ng/mL, p > 0.05). (C) Differential expression of LL-37 and (D) Dsg1 values after 3 M of treatment. LL-37 mRNA expression changes significantly after 3-months of treatment (Newly diagnosed patients_mean_ = 0.039 ± 0.014 vs. 3 M treated_mean_ = 0.408 ± 0.24, p = 0.023) with a notable negative correlation between Dsg1-Abs level and LL-37 mRNA expression. (E) Differential expression level of LL-37 in chronic PV patients (n = 8) compared to remittent (n = 6) and newly diagnosed; (remittent PV_mean_ = 0.049 ± 0.011 vs. Chronic PV_mean_ = 0.225 ± 0.073, p = 0.039). (F) LL-37 mRNA expression changes in chronic PF patients (n = 6), compared to remittent (n = 8); (remittent PF_mean_ = 0.007 ± 0.003 vs. chronic PF_mean_ = 0.074 ± 0.034, p = 0.028; respectively) and newly diagnosed groups (p = 0.013). Comparison of two independent samples using the non-parametrical Mann-Witney test, p-value < 0.05.

To understand the impact of short-term corticosteroid therapy on LL-37 gene expression, six newly diagnosed patients were followed after 3-months of treatment. LL-37 mRNA expression changes significantly after 3-months of treatment (p = 0.023) (Fig. 2C). Interestingly, a notable negative correlation was revealed between Dsg1-Abs level and LL-37 mRNA expression (*r* = -0.7; p = 0.020) (Fig. 2D).

By stratifying PV and PF patients according to their clinical disease stage, LL-37 gene expression was followed up in a group of six-year average treatment period. A notable downregulation of LL-37 expression was identified in remittent group compared to the chronic group (Remittent group_mean_ = 0.025 ± 0.077 vs. Chronic group_mean_ = 0.172 ± 0.05, p = 0.006). A similar profile was observed when studying LL-37 serum expression, without any statistical significance (Remittent group_mean_ = 9.57 ± 1.8 vs. Chronic group_mean_ = 14.24 ± 1.7, p > 0.05) (Fig. 2B). This significance was maintained even when studying the two separate class of pemphigus (PV: p = 0.039 and PF: p = 0.028) (Fig. 2E‒F). There was also a decrease in LL-37 gene expression in remittent PF patients when compared to newly diagnosed ones (p = 0.013).

It seems that the over-expression of LL-37 in peripheral blood cells of newly diagnosed pemphigus patients is important in the disease onset. Yet, there is a lack of studies analysing the LL-37 expression on a systemic level; particularly on PBMC. An up-regulation was largely reported in various biological specimens. Previous studies showed similar results in inflammatory skin disorders.^3^ On the other hand, salivary concentration of LL-37 was increased in inflammatory ulcerating diseases in both oral lichen planus.^4^ Taken together, cathelicidin expression dysregulation appears to occur in pemphigus; PF and PV. However, the precise functions of LL-37 in pemphigus remain elusive. Indeed, both pro- and anti-inflammatory functions have been assigned to LL-37 in a dependent manner on the microenvironment and disease background. Actually, some studies have speculated that these AMPs may provide a protective effect from cutaneous infection in cutaneous lupus erythematosus patients.^3^ However, a pro-inflammatory phenotype of LL-37 on macrophages in systemic lupus was also suggested.^5^

The cornerstone of pemphigus treatment remains systemic corticosteroids due to their immunosuppressive and anti-inflammatory properties.^6^ So, it seems logical to assume that corticosteroids impact the LL-37 expression according to the disease context. The 3-months follow-up showed a significant up-regulation in LL-37 gene expression, which was negatively correlated with anti-Dsg1 Abs in pemphigus monitored patients. Interestingly, chronic pemphigus groups exhibited a persistence of LL-37 high levels in comparison to the remittent ones. Previous research indicated that the glucocorticoid promotes the production of LL-37 and β-defensin.^7^ Actually, corticosteroids play a critical role in remission induction; their interaction with the cytoplasmic corticosteroid receptor results in up-regulation of anti-inflammatory proteins and downregulation of those pro-inflammatory.^6^ Therefore, LL-37 up-regulation may contribute to suppressing the inflammatory responses and mediate tissue repair. Indeed, LL-37 contributes to modulating cytokine production and chemoattracting various immune effector cells, leading to stimulation of angiogenesis and wound healing.^8^ Alongside our results, the up-regulation of LL-37 expression was suggested to be implicated in tissue repair in the recovery phase of sepsis.^9^

On the other hand, there was evidence that LL-37 can perform two distinct functions in different tissues and different microenvironments. This peptide has been shown to regulate monocyte/macrophage differentiation^10^ which can exhibit pro- and anti-inflammatory properties depending on the stimuli from their local microenvironment. In fact, LL-37 exacerbated LPS-induced septic shock in rats when administered 2-hs after LPS treatment.^8^ This is in line with the hypothesis suggesting that the persistence of LL-37 high levels in chronic pemphigus can aggravate the damaging effects induced by inflammation. Thus, the timing and the cellular context could change the LL-37 expression according to the disease severity. On the one side, LL-37 seems to promote immune response and exerts its anti-inflammatory and wound healing effects for remission induction and persistence; on the other side, it seems to have the ability to stimulate inflammation and promote a pro-inflammatory response in the persistent chronic phase of pemphigus. In conclusion, the cellular environment and the timing appear to have an impact on LL-37 expression. For a more comprehensive understanding, further functional studies on skin cell culture are needed.

## ORCID ID

Nesrine Elloumi: 0000-0002-6865-0769

Khadija Sellami: 0000-0002-1565-9663

Emna Bahloul: 0000-0001-6888-2263

Safa Tahri: 0000-0002-3136-0047

Hamida Turki: 0000-0003-0167-6718

Hend Hachicha: 0000-0002-5819-2899

Olfa Abida: 0000-0003-0208-145X

## Ethical approval

The study was approved by the Human Research Ethics Committee of the Habib Bourguiba University Hospital of Sfax (protocol number of the ethical committee, 4/12).

## Financial support

None declared.

## Authors' contributions

Fatma Dhaffouli: Study conception and design; data collection; analysis and interpretation of results and draft manuscript preparation.

Nesrine Elloumi: Study conception and design; analysis and interpretation of results and draft manuscript preparation.

Khadija Sellami: Data collection.

Emna Bahloul: Data collection.

Safa Tahri: Data collection.

Hamida Turki: Data collection.

Hend Hachicha: Data collection.

Olfa Abida: Study conception and design; Analysis and interpretation of results and draft manuscript preparation.

## Research data availability

The entire dataset supporting the results of this study was published in this article.

## Conflicts of interest

None declared.
